# Re: Neglected Major Causes of Death Much Deadlier Than COVID-19

**DOI:** 10.34172/jrhs.2020.17

**Published:** 2020-06-20

**Authors:** Koroush Holakouie-Naieni, Shahrzad Nematollahi

**Affiliations:** ^1^Department of Epidemiology & Biostatistics, School of Public Health, Tehran University of Medical Sciences, Tehran, Iran; ^2^Men`s Health & Reproductive Health Research Center, Shahid Beheshti University of Medical Sciences, Tehran, Iran

## Dear Editor-in-Chief

We reviewed the editorial entitled: "Neglected Major Causes of Death Much Deadlier Than COVID-19", by Prof. Jalal Poorolajal ^1^ with great interest. To answer to the question raised by the author as: "why do people not pay attention to the major causes of death as much as they do to COVID-19?” we grasp the opportunity to reshape the question as:" Why do health policymakers not pay attention to the major causes of death?".

Indeed, the burden of non-communicable diseases must be taken into account. We acknowledge the critical role of communities in prevention and control of diseases, especially non-communicable ones. Meanwhile, we shall not forget that a large portion of prevention and control efforts relies on the governments as a political obligation. There has been a growing awareness of the serious burden of major non-communicable diseases including cardiovascular diseases, diabetes, malignancies, and chronic obstructive pulmonary disease worldwide. In many parts of the world; however, these diseases are being overlooked in prevention measures. Global and national policies have failed to stop, and in many cases have contributed to, the chronic disease pandemic^2^. One example is the critical role of policy makers in nutrition, a significant contributor to many major chronic diseases^3^. Uncoordinated, uninformed policymaking has caused serious consequences to the pattern of food preparation and distribution, leaving insurmountable barriers to make a healthy diet for many people worldwide. These barriers, which are responsible for health inequities, can be overcome thoughtfully by governments to provide evidence-informed policies ^4^. Another example is the governmental contribution in regulating national policies to reduce the amount of sodium intake, another common and well-known risk factor for chronic diseases in most parts of the world^5,6^. Low-cost and highly effective solutions for the prevention of chronic diseases are readily available; the failure to respond is now a political, rather than a technical issue ^2,6^.

Regarding the COVID-19 pandemic, we agree with the author on the important role of communities to stop spreading the pandemic. Nevertheless, the same premise concerning non-communicable diseases exists. Prevention and control of widespread epidemics such as COVID-19 is a political priority and commitment rather than a social responsibility. We disagree with the author on the points that more deaths will occur by the COVID-19, simply because communities will fed up with restrictions. Along with WHO, we truly believe that control of an epidemic or any other health crisis is the foremost priority for governments^7^. As for the COVID-19 epidemic in Iran, despite a few community-based endeavors by the government to tackle the disease^8^, major challenges to combat the epidemic in the country are insufficient whole-government, whole-society approach in managing the outbreak, inadequate lifesaving and protective equipment, and delayed decisive governance^9^. What we have learned from the COVID-19 pandemic shows that successful prevention and control measures relies heavily on the community involvement. Conditional on being asked by policy makers, people are appreciable collaborators in their own health.

In conclusion, we believe that prevention and control of the major causes of death worldwide are indeed health policy priorities and require professional advocacy attempts through appropriate inter-sectoral collaboration and whole-government coalitions. Meanwhile, we should not forget the significant role of media on a thriving community. The premise of a united society, which has a substantial responsibility on its health, is not achievable without governmental efforts to educate, aware, and mobilize it.

## Conflict of interest

The authors declare that there is no conflict of interests.
