# Supplementary material for: Re: Neglected Major Causes of Death Much Deadlier Than COVID-19
Source: J Res Health Sci. 2020 Jun 20;20(2):e00482. doi: 10.34172/jrhs.2020.17 (PMC7585753; doi:10.34172/jrhs.2020.17)
Supplement: Related texts

# Reply: Re: Neglected Major Causes of Death Much Deadlier Than COVID-19

**Published:** 2020-06-20

**Authors:** Jalal Poorolajal

**Affiliations:** ^1^Research Center for Modeling Noncommunicable Diseases and Department of Epidemiology, School of Public Health, Hamadan University of Medical Sciences, Hamadan, Iran

## Reply

I thank very grateful to Prof. Koroush Holakouie-Naieni and his colleague for their kind comments on my recent editorial in the Journal of Research in Health Sciences.

I appreciate their concern and emphasize that their question “Why do health policymakers not pay attention to the major causes of death?” and mine “Why do people not pay attention to the major causes of death as much as they do to COVID-19?” are the both sides of the same coin. Indeed, people's lack of attention to the major causes of death and dangerous diseases is the result of negligence of the health policy makers and media. As I emphasized in the last paragraph of the paper, people do not pay enough attention to the importance and catastrophic consequences of common causes of death because they hear little about these realities in media ^1^. On the other hand, COVID-19 has become the major concern of the general population for several months because a huge propaganda and international campaign was launched to curb the disease. The media has a remarkable impact on public beliefs and awareness ^2^. They can manage and shape public beliefs and attitudes ^3, 4^. So people pay attention to the same things on which policymakers and media focus.

## References

[1] Poorolajal J (2020). Neglected major causes of death much deadlier than COVID-19. J Res Health Sci.

[2] Laker-Oketta M, Butler L, Kadama-Makanga P, Inglis R, Wenger M, Katongole-Mbidde E (2020). Using media to promote public awareness of early detection of Kaposi's Sarcoma in Africa. J Oncol.

[3] Oto MA, Ergene O, Tokgozoglu L, Ongen Z, Kozan O, Sahin M (2011). Impact of a mass media campaign to increase public awareness of hypertension. Turk Kardiyol Dern Ars.

[4] Gollust SE, Attanasio L, Dempsey A, Benson AM, Fowler EF (2013). Political and news media factors shaping public awareness of the HPV vaccine. Women's Health Issues.
